# Management of chondral and osteochondral lesions of the hip: A comprehensive review

**DOI:** 10.1007/s00132-023-04444-9

**Published:** 2023-10-10

**Authors:** Rajesh Itha, Raju Vaishya, Abhishek Vaish, Filippo Migliorini

**Affiliations:** 1 Department of Orthopaedics, ESIC Model Hospital, 201307 Noida, Uttar Pradesh, India; 2 Department of Orthopaedics and Joint Replacement Surgery, Indraprastha Apollo Hospital, Sarita Vihar, 110076 New Delhi, India; 3 Department of Orthopaedic, Trauma, and Reconstructive Surgery, RWTH University Medical Center of Aachen, 52064 Aachen, Germany; 4 Department of Orthopedics and Trauma Surgery, Academic Hospital of Bolzano (SABES-ASDAA), 39100 Bolzano, Italy

**Keywords:** Cartilage, Subchondral, Impingement, Microfracture, Chondroplasty, Hyaluronic Acid, Prosthetic Biocomposites, Autograft, Allograft, Knorpel, Subchondral, Einklemmung, Mikrofrakturierung, Chondroplastie, Hyaluronsäure, Prothetische Biokomposite, Autograft, Allograft

## Abstract

Chondral and osteochondral lesions encompass several acute or chronic defects of the articular cartilage and/or subchondral bone. These lesions can result from several different diseases and injuries, including osteochondritis dissecans, osteochondral defects, osteochondral fractures, subchondral bone osteonecrosis, and insufficiency fractures. As the cartilage has a low capacity for regeneration and self-repair, these lesions can progress to osteoarthritis. This study provides a comprehensive overview of the subject matter that it covers. PubMed, Scopus and Google Scholar were accessed using the following keywords: “chondral lesions/defects of the femoral head”, “chondral/cartilage lesions/defects of the acetabulum”, “chondral/cartilage lesions/defects of the hip”, “osteochondral lesions of the femoral head”, “osteochondral lesions of the acetabulum”, “osteochondral lesions of the hip”, “osteochondritis dissecans,” “early osteoarthritis of the hip,” and “early stage avascular necrosis”. Hip osteochondral injuries can cause significant damage to the articular surface and diminish the quality of life. It can be difficult to treat such injuries, especially in patients who are young and active. Several methods are used to treat chondral and osteochondral injuries of the hip, such as mesenchymal stem cells and cell-based treatment, surgical repair, and microfractures. Realignment of bony anatomy may also be necessary for optimal outcomes. Despite several treatments being successful, there is a lack of head-to-head comparisons and large sample size studies in the current literature. Additional research will be required to provide appropriate clinical recommendations for treating chondral/osteochondral injuries of the hip joint.

## Abstract (de)

Chondrale und osteochondrale Läsionen umfassen verschiedene akute oder chronische Defekte des Gelenkknorpels und/oder des subchondralen Knochens. Diese Läsionen können durch viele unterschiedliche Erkrankungen und Verletzungen verursacht sein, darunter Osteochondritis dissecans, osteochondrale Defekte, osteochondrale Frakturen, subchondrale Knochennekrose und Insuffizienzfrakturen. Da Knorpel eine geringe Fähigkeit zur Regeneration und Selbstheilung hat, können die Läsionen zur Arthrose fortschreiten. Die vorliegende Studie bietet einen umfassenden Überblick zum Thema. PubMed, Scopus und Google Scholar wurden mit den folgenden Begriffen durchsucht: „chondral lesions/defects of the femoral head“, „chondral/cartilage lesions/defects of the acetabulum“, „chondral/cartilage lesions/defects of the hip“, „osteochondral lesions of the femoral head“, „osteochondral lesions of the acetabulum“, „osteochondral lesions of the hip“, „osteochondritis dissecans“, „early osteoarthritis of the hip“ und „early stage avascular necrosis“. Osteochondrale Verletzungen der Hüfte können einen erheblichen Schaden an der Gelenkoberfläche verursachen und die Lebensqualität verringern. Die Behandlung solcher Verletzungen kann sich schwierig gestalten, insbesondere bei jungen und aktiven Patienten. Zur Therapie chondraler und osteochondraler Verletzungen der Hüfte werden verschiedene Verfahren angewendet, so etwa Behandlungen mit mesenchymalen Stammzellen und zellbasierte Therapien, operative Eingriffe und Mikrofrakturierung. Auch ein Realignment der Knochenanatomie kann für optimale Behandlungsergebnisse nötig sein. Trotz mehrerer erfolgreicher Therapieverfahren fehlt es in der aktuellen Literatur an Direktvergleichen und Studien mit großen Stichproben. Weitere Studien sind erforderlich, um angemessene klinische Empfehlungen für die Behandlung chondraler bzw. osteochondraler Verletzungen des Hüftgelenks formulieren zu können.

## Introduction

Chondral and osteochondral lesions encompass several acute or chronic defects of the articular cartilage and/or subchondral bone. The chondral lesions are located solely on the cartilage surface, whereas the osteochondral lesions are located in both cartilage and subchondral bone. Goyal et al. compared the subchondral bone-cartilage equilibrium to the soil-plant equilibrium. Soil provides plants with nutrients, provides a stable environment for their roots to grow in, and these roots of plants prevent soil erosion. The subchondral bone acts as rich soil for cartilage and bears its loads [1]. Damage to various tissues in the joint, including the subchondral bone below, may result from, be caused by, or happen simultaneously with damage to the articular surface [2]. These lesions arise from a wide range of pathologies, such as femoroacetabular impingement, developmental dysplasia of the hip, osteochondritis dissecans, osteochondral defects, osteochondral fractures, subchondral bone osteonecrosis, and insufficiency fractures [3]. Osteochondral lesions can be generated by both traumatic and atraumatic conditions damaging the cartilage and subchondral bone [4]. Chondral lesions cannot heal themselves completely. Due to the migration of bone marrow mesenchymal cells (BM-MSC) and the development of an inflammatory “super clot,” full-thickness lesions with subchondral bone involvement can heal to some extent. The freshly formed fibrocartilage tissue has a different structure to the initial hyaline articular cartilage. It is mostly made of type I collagen, while hyaline cartilage is mostly made of type II collagen [5]. These lesions frequently advance to osteoarthritis (OA), which is regarded as “an organic disease of the whole joint,” because cartilage has limited ability for regeneration and self-repair [2, 4]. Hip chondral lesions continue to be challenging to diagnose and treat for orthopedic surgeons. Imaging technology, arthroscopic equipment, and insights from fundamental science and clinical research have contributed to a substantial increase in hip arthroscopy procedures over the past decade. These factors have led to a rise in the detection and treatment of hip chondral lesions [6–8]. With the development of numerous technologies, the idea of joint preservation was introduced to avoid or slow the onset of osteoarthritis as well as to preserve or restore joint function in joints already afflicted by osteoarthritis. Over the last decade, intriguing innovative techniques based on novel tissue engineering techniques have been developed to address chondral/osteochondral lesions of the hip [4]. The current study discusses a comprehensive overview of the management of the osteochondral lesions hip, various pathological processes associated with the osteochondral lesions hip and the currently available treatment options.

## Functional anatomy of the hip joint

The hip functions as a ball and socket joint during stance and walking to keep the torso balanced. The congruency of the articulating surfaces precludes femoral head and acetabulum translation. Strong articular congruency is provided by bone cartilage, the acetabular labrum, articular cartilage, the inner capsule, and surrounding musculature [9]. The cotyloid fossa comprises a combination of fibrofatty tissue and synovium lining. The depression of the cotyloid extends into the acetabular fossa. The articular surface of the acetabulum is an upside-down, cartilage-covered horseshoe. Hyaline cartilage covers the femoral head, excluding the fovea capitis femoris, a depression on the femoral head. This depression gives rise to the ligamentum teres femoris, which attaches medially to the transverse ligament and other tissues [10]. The articular surface of normal hips has variable hyaline cartilage thickness. The average cartilage thickness in the acetabulum is 3 mm, but it can vary between 1.5 and 5 mm. The deepest point of the cartilage in the centre of the femoral head ranges from 1.5 to 5 mm in thickness. The cartilage at the femoral head’s periphery has an average thickness of 1 mm, whereas the cartilage in the anterior, superior, and medial regions of the acetabulum has an average thickness of 1.3 mm [11, 12].

## Osteochondral unit

Several components make up the articular joint, each playing a crucial role in its proper function. These elements include articular cartilage, bone, synovium, ligaments, capsule and labrum. The joint performs the critical functions of providing smooth mobility and weight-bearing support. Articular cartilage, in particular, is essential for these functions, and its homeostasis is maintained by the subchondral bone. The term osteochondral unit refers to this harmonious relationship that exists between articular cartilage and subchondral bone in a joint which is essential for both the weight bearing and the mobility of a joint. Preservation of this unit is necessary for joint health as osteochondral injury and degeneration can impair joint function. While some treatment techniques focus solely on repairing articular cartilage, subchondral bone must also be repaired for successful outcomes, because it serves a crucial part in the normal functioning of joint cartilage [13].

## Histology

**Fig. 1:**
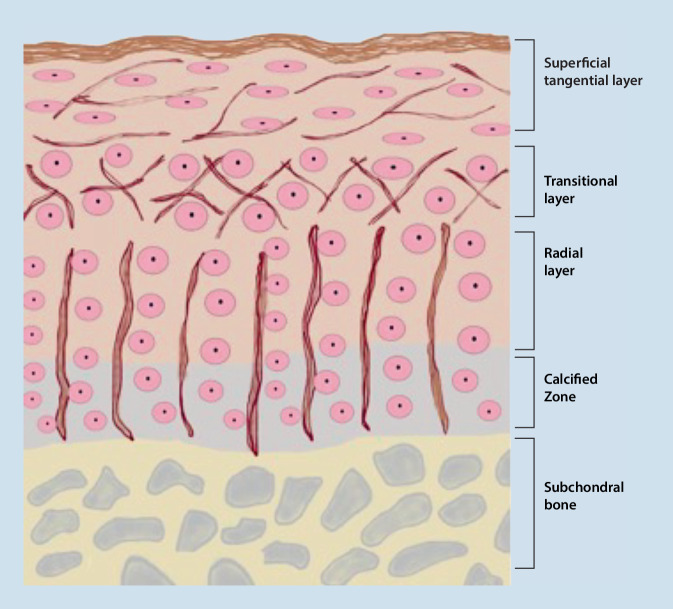
Illustration of the chondral layers

The joint contains the vital osteochondral unit consisting of hyaline cartilage and subchondral bone. Chondrocytes, which are responsible for cartilage metabolism, synthesize and degrade proteoglycans and collagens within the unit, which has four distinct layers across multiple zones. The radial zone, constituting the majority of the articular cartilage, boasts a well-developed rough endoplasmic reticulum and Golgi apparatus, while a tidemark separates it from the calcified zone. The subchondral osteochondral bone, made up of metaphyseal trabecular bone, has small holes through which blood vessels penetrate the calcified layer. It effectively absorbs loads, enabling the transmission and distribution of the cartilage matrix [14]. A schematic representation of the chondral layers is shown in Fig. 1.

## Physiology and pathophysiology

The joint cartilage and the subchondral bone that form the osteochondral unit maintain the joint’s equilibrium. In normal conditions only 1–3% of the load is absorbed by cartilage but microfractures and other damage caused by FAI or dysplasia of the hip can lead to abnormal remodelling and a loss of its shock-absorbing ability, resulting in cartilage degeneration. Cartilage is nourished by two main methods: diffusion through synovial fluid in the superficial layer and vascularity in the deep calcified layer. The canalicular/lacunar network transports larger molecules, which are required for appropriate cartilage nourishment and repair [15].

## Pathogenesis of chondral/osteochondral lesions

The pathogenesis of chondral and osteochondral lesions involves a complex interplay of mechanical, biochemical, and cellular factors. The initial insult often involves a traumatic event, such as a sports injury or a sudden impact, which causes damage to the cartilage and underlying bone [16, 17]. An injury to the chondral tissue of the hip joint can be caused by damage to the acetabular labrum. By stopping the leakage of joint fluid and functioning as a blocking mechanism for the interstitial fluid content, the labrum plays an essential part in the preservation of the structural integrity of the joint [18]. This trauma disrupts the smooth and frictionless surface of the cartilage, leading to the release of inflammatory mediators and activation of various cellular processes. Over time, the damaged cartilage undergoes degeneration and loss, resulting in compromised joint function. Furthermore, the altered biomechanics and increased stress on the affected area contribute to the development of osteoarthritis [16, 17].

## Etiology

Chondral and osteochondral lesions in the hip can result from a variety of conditions, including femoroacetabular impingement (FAI), developmental dysplasia (DDH), avascular necrosis (AVN) and osteochondritis dissecans (OCD), and joint infection, rheumatic disease. Traumatic injuries such as hip joint dislocation, femoral head fracture, acetabular fracture or osteoarthritis can also cause these lesions.

FAI is one of the hip disorders caused by cam or pincer deformities. Cam impingement damages the anterosuperior and lateral acetabulum, while pincer lesions cause circumferential damage to the acetabular cartilage. FAI can also be caused by version abnormalities of the acetabulum or femur. The abnormal contact between the femoral head and the acetabulum in FAI results in mechanical stress on the articular cartilage, impaired blood flow, an inflammatory response, and altered joint biomechanics. These factors contribute to chondral damage, including fissuring, delamination, cartilage fibrillation, and the formation of osteochondral lesions. Understanding the mechanisms by which FAI produces these lesions is essential for appropriate management [19, 20].

In DDH, the shallow acetabulum fails to adequately cover and support the femoral head, leading to increased stress concentration on weight-bearing regions of the articular cartilage. This abnormal biomechanics cause repetitive microtrauma and friction between the femoral head and acetabulum, resulting in chondral damage. The instability of the hip joint in DDH further increases the risk of chondral and osteochondral lesions due to excessive movement and subluxation. These abnormal movements generate shear forces and impact stresses on the articular cartilage, leading to chondral injuries. Repeated subluxation or dislocation events can also cause osteochondral lesions, affecting both the cartilage and underlying bone. The presence of chondral and osteochondral lesions perpetuates joint instability, deformity, and abnormal loading, progressing the disease [21, 22].

In slipped capital femoral epiphysis (SCFE), the altered biomechanics due to femoral head displacement cause abnormal stress and shear forces within the hip joint. These forces can damage the articular cartilage, leading to chondral lesions. Additionally, the femoral head displacement can disrupt blood supply, resulting in avascular necrosis (AVN), which leads to bone and cartilage damage, causing osteochondral lesions. The development of chondral and osteochondral lesions in SCFE is influenced by factors such as slip severity, duration, and patient characteristics [23, 24].

Legg-Calvé-Perthes (LCP) disease, a pediatric hip disorder, leads to chondral and osteochondral lesions. It involves disrupted blood supply to the femoral head, causing AVN and structural changes. Ischemia leads to bone cell death, microfractures, and resorption. Revascularization occurs, but the regenerated bone may be weak and prone to fractures. Altered biomechanics and irregularities in the femoral head result in cartilage thinning and fibrillation. Abnormal contact pressures cause further cartilage damage and osteochondral lesions. Early diagnosis and management are vital to minimize long-term effects [25–27].

In the case of AVN, the compromised blood supply can lead to the death of osteocytes, which are the bone cells responsible for maintaining the structure and integrity of the bone tissue. The loss of osteocytes weakens the affected bone, making it more prone to damage. The progression of AVN involves the formation of microfractures within the necrotic bone. These microfractures disrupt the continuity of the bone structure and can extend to involve the overlying articular cartilage. As a result, chondral and osteochondral lesions can develop. The mechanical stress placed on the compromised bone and cartilage can further contribute to the development of chondral and osteochondral lesions. The altered biomechanics and increased load-bearing demands on the affected joint can lead to cartilage degeneration and wear. Over time, this can result in the loss of articular cartilage, exposing the underlying bone and leading to the formation of osteochondral lesions. It is important to note that AVN and its association with chondral and osteochondral lesions can vary depending on the specific location and extent of the AVN, as well as individual patient factors [28–30].

In the hip joint, OCD is very rare but it can produce chondral and osteochondral lesions through several mechanisms. The initial insult often involves repetitive trauma or microtrauma to the joint, which disrupts the blood supply to the subchondral bone and overlying cartilage. This compromised blood flow can lead to ischemia, resulting in the degeneration and weakening of the affected area. As a result, the affected cartilage and underlying bone become susceptible to damage. The mechanical forces exerted on the hip joint during weight-bearing activities further contribute to the development of chondral and osteochondral lesions. Over time, the weakened area can undergo further degeneration, leading to the detachment of a fragment of cartilage and bone [31, 32].

## Classification

Chondral damage can be categorized in different ways, which, along with the size of the damage, can help figure out the best way to treat it. Outerbridge’s classification system, developed in 1961, is based on the severity of cartilage disruption and is widely used to grade chondral lesions [33]. Beck et al. developed a modified Outerbridge’s classification system that includes a grade 0 for normal cartilage and adds subgrades to grade III [34]. The classification system of the International Cartilage Repair Society (ICRS) classifies lesions according to their appearance, location, and depth. The appearance and location can be determined with MRI and X-ray, but the depth can only be determined with intraoperative findings [35]. Konan et al. proposed an expanded classification system that includes the six acetabular zones defined by Ilizaliturri et al. and the size of the lesion. This system is particularly useful for diagnosing and treating FAI pathology [36]. Additionally, Sampson created two classification systems for cartilage lesions, one for the femoral head and the other for the acetabulum, and suggested treatment protocols based on these classifications [37]. Table 1 describes various classifications used for chondral lesions.

**Table 1:** Various classification systems for categorization of chondral/osteochondral lesions of hip jpoint.

Classification	Grade/type	Description	Structures involved
Outerbridge [33]	0	Normal cartilage	–
	1	Cartilage is soft and swollen	–
	2	Partial thickness lesion with a diameter less than 0.5 inches	–
	3	Partial thickness lesion with a diameter greater than 0.5 inches	–
Beck [34]	0	Normal	–
	1	Softening of the cartilage	–
	2	Separation of cartilage from bone	–
	3	Cartilage tear with fibrillation	–
	4	Full thickness cartilage defect reaching the subchondral bone	–
International Cartilage Repair Society (ICRS) [35]	0	Normal	–
	1	Nearly normal: minor surface irregularities	–
	2	Abnormal: lesions involving no more than 50% of cartilage thickness	–
	3	Severely abnormal: lesions affecting more than 50% of cartilage thickness	–
	4	Severely abnormal: lesions extending into the subchondral bone	–
Konan [36]	I	Focal articular cartilage lesion	–
	II	Partial thickness lesion	–
	III	Full-thickness lesion	–
Modified Konan [36]	I	Focal articular cartilage lesion	Articular cartilage
	IIa	Partial thickness lesion (< 50% depth)	Articular cartilage and subchondral bone
	IIb	Partial thickness lesion (> 50% depth)	Articular cartilage and subchondral bone
	IIIa	Full-thickness lesion (< 50% of surface area)	Articular cartilage and subchondral bone
	IIIb	Full-thickness lesion (> 50% of surface area)	Articular cartilage and subchondral bone
	IV	Full-thickness lesion with cyst formation	Articular cartilage and subchondral bone
	V	Flap lesion (detached fragment of cartilage and subchondral bone)	Articular cartilage and subchondral bone
	VI	Degenerative joint disease	Articular cartilage, subchondral bone, and joint space
Acetabular labrum articular disruption (ALAD) [36]	1	Softening of the adjacent cartilage	–
	2	Early cartilage peeling back	–
	3	A sizable cartilage separation as flap	–
	4	Loss of cartilage	–

**Table 2:** Summary of the biological treatment methods for chondral/osteochondral lesions in the hip

Treatment	Description	Effectiveness	Advantages	Disadvantages
Hyaluronic acid (HA) injections	Injection of hyaluronic acid to promote cartilage regeneration and joint function	Substantial relief from symptoms and aid in repairing and restoring damaged areas of the hip joint	Low cost and minimal risks	May not provide a cure for hip chondral lesions
Platelet-rich plasma (PRP)	Treatment that uses high concentration of growth factors and cytokines found in platelets to promote tissue healing and regeneration	Promising results reported, low cost and minimal risks	Reduces the inflammatory environment associated with OA	Inconsistent outcomes may be influenced by factors such as patient demographics, preparation methods, and PRP constituent concentration
Mesenchymal stem cells (MSC) and bone marrow aspirate concentrate (BMAC)	Injection of stem cells from bone marrow aspirate to promote cartilage regeneration	Significant improvements in clinical outcomes reported, successful cartilage regeneration and improvement in clinical scores	High concentration of MSCs, potentially leading to better clinical results	Optimal dose, frequency, and number of injections are still uncertain

**Table 3:** Summary of the surgical treatment for chondral/osteochondral lesions of the hip joint

Technique	Description	Indications	Success factors	Limitations
Chondroplasty	Smoothing of unstable chondral flaps, removing mechanical blocks	Low-grade and partial-thickness chondral injuries	Good clinical outcomes for low-grade injuries	Increased likelihood of conversion to total hip replacement in some cases; not suitable for advanced arthritis
Repair of chondral flap	Surgical repair of delaminated cartilage flap with sutures	Delaminated cartilage flaps	Improved patient outcomes reported in studies	Limited studies on efficacy; requires further investigation
Microfracture	Creation of small holes in bone to stimulate fibrocartilage formation	Osteochondral lesions	Size and location of lesion, patient’s age, and activity level	Potential risks such as ossification, fragility of tissue, and inadequate filling of lesion
Augmented microfracture	Use of scaffolds or growth factors to enhance microfracture repair	Osteochondral lesions	Improved quality of repair tissue reported in studies	Longer culture times and complex preparation may contribute to failure
ADSCs and microfragmented adipose tissue transplantation (MATT)	Transplantation of adipose-derived stem cells or microfragmented adipose tissue	Small acetabular chondral defects	Easier to isolate and higher proliferation rate compared to other stem cells	Limited studies, further research needed
Autologous chondrocyte implantation (ACI)	Harvesting chondrocytes for implantation with a bioabsorbable matrix	Large cartilage defects	Improved patient outcomes reported in some cases	Difficulties with harvest and surgical procedure on unaffected joint
Autologous matrix-induced chondrogenesis (AMIC)	Use of collagen matrix with microfracture to treat Grade 3–4 defects	Grade 3–4 chondral defects	Increased patient activity level and pain reduction reported in studies	Long-term benefits and risks require further evaluation
Three-dimensional (3D) ACI	Culture of autologous chondrocytes into 3D spheroids for injectable solution	Medium to large articular cartilage defects	Improved patient outcomes and cartilage healing reported in studies	Longer culture times and complex preparation may contribute to failure
Autologous minced cartilage implantation (AMCI)	Arthroscopic cartilage preparation, mincing with ACP, implantation	Acetabular lesions in FAIS patients	Cartilage repair, own tissue use	Arthroscopic, variable efficacy, long-term unknown
Osteochondral autograft transplantation (OAT) and mosaicplasty	Transplantation of osteochondral plugs from non-weight-bearing surface	Larger or multiple defects, failed microfracture	Enhances clinical outcomes and range of motion	Risk of subsequent hip arthroscopy with mosaicplasty
Osteochondral allograft transplantation (OCA)	Replacement of damaged joint surface with allograft	Large and difficult-to-treat defects	Instant functional joint surface and potential replacement of hyaline cartilage	Challenges with donor tissue matching, timing, and limited supply
Prosthetic biocomposites	Use of synthetic scaffolds for tissue regeneration	Osteochondral defect repair	Potential for guiding tissue regeneration	Challenges in achieving anatomical and biomechanical stratification

## Clinical assessment

A comprehensive clinical assessment is essential in the diagnosis of hip disorders and associated chondral/osteochondral injuries. The clinical manifestations of hip disorders such as FAI, dysplasia, osteochondritis dissecans, and AVN femoral head that cause chondral injury and hip discomfort vary. Unfortunately, patients with FAI wait an average of 4.2 months before seeking medical attention, and 3 years before receiving a diagnosis. In addition, approximately 13% of patients undergo operations that fail to treat the underlying hip disorder. Importantly, chondral injuries may be a consequence rather than a cause of the hip disorder [38]. Patient medical history, including prior injuries, hip conditions during childhood, athletic activities, and past surgical procedures, provides valuable insights into potential underlying causes and influences treatment options. A thorough physical examination is performed, including range of motion assessment, provocative tests, palpation, and neurovascular evaluation. These evaluations aid in identifying associated symptoms, joint instability, and mechanical issues [39].

## Radiological assessment

**Fig. 2:**
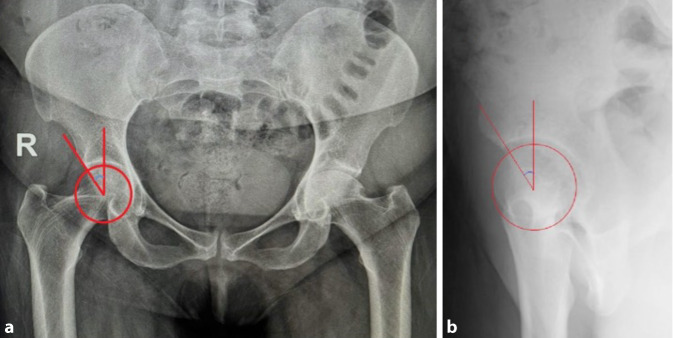
**a** AP pelvic radiograph shows LCEA on the right hip; The LCEA is the angle between the vertical line from the femoral head center and the line connecting the lateral margin of the acetabulum. **b** The right hip false profile radiograph shows ACEA; The ACEA is the angle between the vertical line from the center of the femoral head and the posterior margin of the acetabulum.

The hip joint is evaluated using a variety of imaging techniques. The acetabular index (AI) and lateral center-edge angle (LCEA) are essential parameters to take into account when interpreting anteroposterior (AP) pelvic radiographs ([40]; Fig. 2a). False profile radiography helps evaluate posterior degenerative joint changes and anterior femoral head coverage, which can be measured by calculating the anterior centre-edge angle (ACEA) ([41]; Fig. 2b).

**Fig. 3:**
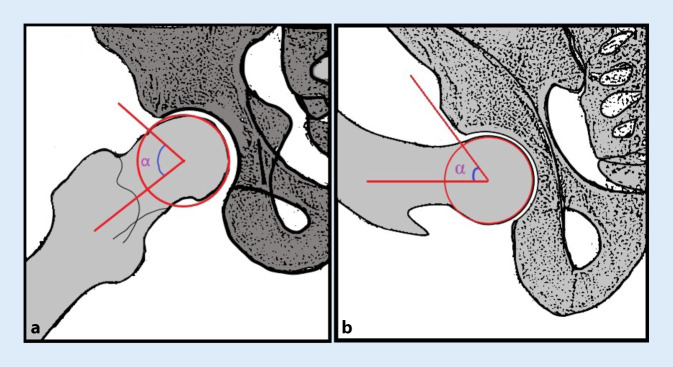
Diagrammatic representation of radiographs of 45° Dunn’s view (**a**) and a 90° Dunn’s view (**b**). The alpha angle is formed by two lines. One line connects the center of the femoral neck’s long axis to the center of the femoral head. Another line goes from the center of the femoral head to the location on the anterolateral head-neck junction. This is the point where the radius of the femoral head starts to increase beyond the radius that is typically found more centrally in the acetabulum, where the head is more spherical. It is a measure of the asphericity of the femoral head and neck

The Dunn view is commonly employed to evaluate the sphericity of the femoral head in patients suspected of having FAI ([42]; Fig. 3). The alpha angle measures cam lesions, but other anatomical parameters can also affect clinical significance [43]. Other radiographic tools such as the cross-table lateral view and the frog-leg lateral view are also useful in evaluating FAI [44]. These tools aid in diagnosing and treating conditions affecting the hip joint.

CT is a highly effective imaging technique for evaluating the alignment of bones and detecting osteochondral injuries around the hip [45]. This method allows for accurate measurement of the extent to which the subchondral bone is involved and assessment of areas of irregularity around the junction of the femoral head and neck [46]. When combined with three-dimensional reformatting, CT provides an advantage over plain radiography and MRI in identifying both intra-articular and extra-articular impingement, including subspinal impingement [47]; however, it is important to note that while the numerical values of angle measurements used to diagnose abnormalities in coverage are based on plain X‑ray imaging, they do not correspond with the center-edge angle measurements obtained from coronal and sagittal CT slices [48].

Magnetic resonance imaging (MRI) is a most useful nonradiation method for assessing nontraumatic osteochondral pathologies of the hip joint. It can identify injuries to the labrum and areas of bony edema linked to intra-articular impingement. The latest high-field MRI technology can detect abnormalities without the need for contrast agents. To examine and define osteochondral abnormalities, several techniques, such as true proton density and T2-weighted turbo spin-echo are employed. Additionally, advanced techniques like T2 relaxation time and delayed gadolinium-enhanced MRI of cartilage (dGEMRIC) can be used to evaluate cartilage abnormalities [49].

## Treatment of underlying causes

In cases of FAI treatment options range from nonsurgical to surgical interventions. Nonsurgical approaches encompass physical therapy to enhance hip flexibility and strength, injections for inflammation reduction, and activity modification. Surgical treatment includes hip arthroscopy or open hip surgery for decompressing the bony prominence around the rim and at the femoral head neck junction. Optimal treatment depends on FAI severity and patient-specific considerations [50].

The treatment of DDH varies by age and severity. A Pavlik harness is common for infants. Surgical options for older children include closed/open reduction, osteotomy, or arthroscopy, aiming to normalize femoral head coverage and address labral injury. Surgical selection considers age, CE angle, and OA grade for optimal outcomes [51].

Treatment for slipped capital femoral epiphysis (SCFE) primarily involves surgery to stabilize the hip joint and prevent further slippage. Surgical options vary based on the severity: mild cases may require a single screw across the growth plate, while severe cases may involve multiple screws or an osteotomy. After surgery, cast/brace use aids healing, and physical therapy restores hip joint mobility. Recognition of labral injury, hip dysplasia, patient age, CE angle, and OA grade guides surgical treatment selection for optimal outcomes [52].

Surgical intervention is often necessary for young adult patients of Legg-Calvé-Perthes disease with worsening hip pain and dysfunction caused by hip articulation deformities. Treatment may involve proximal femoral osteotomy or pelvis osteotomy to restore normal femoral head coverage. In addition, when performing surgical dislocation with the trochanter, concurrent relative neck lengthening may be considered [27].

To treat osteochondral lesions in AVN of the hip, various approaches are available, including core decompression, osteochondral autograft transplantation, and total hip arthroplasty depending on the Association Research Circulation Osseous (ARCO) classification [53].

Treatment for osteochondritis dissecans (OCD) of the hip varies is based on lesion size, location, patient age, and symptom severity. Rest, physical therapy, and injections can be initial approaches. Surgery is considered if conservative methods fail or for severe cases. Surgical options include microfracture, autologous chondrocyte transplantation (ACT), and osteochondral autograft transplantation (OAT) based on lesion specifics [32].

## Treatment of chondral/osteochondral lesions

It is important not only to treat the chondral/osteochondral lesions but also to treat the underlying cause of the lesion. For example, if the lesion is caused by labral tears, surgery may be needed to repair the labrum and if the lesion is caused by FAI, surgery may be needed to correct the deformity.

Hip chondral/osteochondral lesions can be treated using various methods, which can be broadly categorized into conservative, less invasive approaches and more complex procedures. Treatment decisions depend on factors, such as patient symptoms, lesion size, and activity level. Treatment algorithms have been developed to guide these decisions and provide tailored treatments.

## Conservative

Conservative treatment for chondral/osteochondral lesions of the hip involves nonsurgical approaches aimed at reducing symptoms, promoting healing, and improving joint function. Conservative treatment for chondral/osteochondral lesions of the hip is indicated in cases of mild to moderate symptoms, stable and small lesions, absence of mechanical symptoms, and when the patient prefers nonsurgical options [54]. These options include patient education, pain medication, physical therapy, and muscle strengthening.

- Patient education: providing information and education to patients about their condition, including the nature of the hip pain and strategies for symptom management.
- Symptom control: the use of nonsteroidal anti-inflammatory drugs (NSAIDs) to help reduce pain and inflammation associated with hip pain.
- Identification and modification of aggravating activities: identifying activities that worsen symptoms and modifying or avoiding them to reduce stress on the hip joint.
- Physical therapy interventions: physical therapy programs aimed at addressing neuromuscular deficits, strengthening the hip and lumbopelvic regions, improving core stability, and enhancing flexibility and range of motion. These may include exercises targeting hip musculature, pelvic positioning, core muscle strengthening, neuromuscular training, stretching, manual therapy, dynamic biomechanical control, and gait training.
- Dynamic stabilization: establishing dynamic stabilization of the hip musculature, core, and pelvic regions to prevent excessive hip joint motion during activities.

## Biologics 

Biological treatments provide promising options for managing chondral and osteochondral lesions of the hip by promoting the regeneration of damaged joint tissue (Table 2). Hyaluronic acid (HA) injections have been found to be effective in managing chondral lesions of the hip by facilitating the regeneration of articular cartilage and promoting the healing process. HA injections not only provide lubrication and cushioning but also stimulate the production of chondrocytes, which are crucial for the formation of cartilage. By promoting the growth of new cartilage tissue, HA injections aid in repairing and restoring damaged areas of the hip joint. In cases where conservative treatments have failed to provide relief, HA injections can play a significant role in managing chondral lesions of the hip. Although HA injections may not provide a cure for hip chondral lesions, they can offer substantial relief from symptoms and help enhance joint function and overall quality of life [55–57].

PRP therapy is a non-immunogenic treatment derived from the patient’s own blood, where platelets are concentrated in a small volume of plasma, typically 3–6 times higher than baseline [58]. This therapy offers several advantages, including quick preparation and simplicity in its technique. Being autologous in nature, PRP therapy carries a distinct safety profile, as it lacks many of the side effects and interactions associated with pharmaceutical drugs [59]. PRP has been investigated as a potential treatment option for chondral and osteochondral lesions of the hip, although research in this area is limited. Animal studies have shown promising results with intra-articular injections of PRP and autologous conditioned plasma, as well as the use of platelet-enriched fibrin scaffolds [60]; however, there have been no published studies on the use of PRP for chondral defects in human subjects. The studies which examined the effects of PRP for hip osteoarthritis (OA) showed lower pain scores and better functional outcomes [61, 62].

The distinction between stem cells and bone marrow aspirate concentrate (BMAC) is that stem cells are undifferentiated cells with the ability to differentiate into various cell types, whereas BMAC is the concentration of stem cells, growth factors, and cytokines found in the bone marrow. BMAC contains a high number of stem cells, ranging from 0.001% to 0.01% [63]. To increase stem cell concentrations, they are isolated from bone marrow aspirate, seeded, and expanded for 2–6 weeks [63]. The optimal dose, frequency, and number of injections are still uncertain, but some studies indicate that higher concentrations of stem cells can lead to better clinical results [64]. MSCs can be used in the treatment of osteochondral defects with both reparative and preventative effects [65]. Gobbi et al. (2019) used expanded MSCs to treat chondral defects in 20 patients and reported significant improvements in clinical outcomes and MRI showing good to excellent repair tissue [66]. Centeno et al. (2018) reported better clinical outcomes with BMAC treatment for the treatment of knee osteoarthritis [67]. Based on these studies, stem cells and BMAC may be an effective treatment option for chondral and osteochondral lesions in the hip joint.

## Surgery

Surgical treatments play a crucial role in addressing chondral and osteochondral lesions of the hip, offering a range of techniques and approaches to restore joint function and alleviate symptoms (Table 3). Chondroplasty is a widely used technique in hip arthroscopy that involves the smoothing of areas with unstable chondral flaps (mostly acetabular lesions), preventing the development of loose bodies and removing potential mechanical blocks in the joint. This method is preferred for treating low-grade and partial thickness chondral injuries and has been proven successful in such cases [68]; however, studies have indicated that performing chondroplasty during hip arthroscopy may increase the likelihood of conversion to total hip replacement in patients of all ages [69]. Chondroplasty should not be performed on advanced arthritis that requires total hip arthroscopy and should be preferentially carried out on patients with pre-existing OA [69]. It is also important to avoid using radiofrequency ablation devices around chondral tissue, as they can damage chondrocytes [70]. Chondroplasty is the most commonly performed procedure in hip arthroscopy, accounting for 49.3% of cases [68]. Good clinical outcomes have been observed with chondroplasty, making it a satisfactory treatment strategy for low-grade and partial thickness chondral injuries [68]; however, the decision to perform chondroplasty should be made on a case by case basis, taking into account the patient’s age, overall joint health, and the severity of the injury.

Cartilage delamination flaps can be repaired surgically with sutures. Sekiya et al. performed arthroscopic microfracture and suture repair of delaminated cartilage flap and reported good outcomes measured by modified Harris hip score and hip outcome scores [71]. Tzaveas et al. conducted a study on the efficacy of fibrin adhesive for arthroscopic repair of chondral delamination lesions. They found promising short-term results, with intact chondral repairs in cases that underwent revision arthroscopy [72].

Microfracture is a minimally invasive procedure used to treat osteochondral lesions in the hip. This technique involves the creation of small holes in the affected bone to stimulate the formation of fibrocartilage. The success of microfracture depends on factors such as the size and location of the lesion, as well as the patient’s age and level of physical activity. Although the formation of fibrocartilage is a potential limitation, microfracture remains a viable option for many patients and can be performed on an outpatient basis [73]. Various studies reported good outcomes following microfracture [74, 75]. Microfracture offers several advantages, including its relatively low cost and the fact that it is not considered a technically challenging procedure;however, it is important to consider the potential risks associated with microfracture. These risks include the possibility of ossification, fragility of the newly formed tissue, imperfections in the regenerated cartilage, inadequate filling of the lesion, and the susceptibility of the new cartilage to breakdown over time [76].

The repair tissue formed following microfracture has inferior properties compared to normal hyaline cartilage, leading to concerns about its long-term durability. In order to improve the outcomes of the microfracture procedure, several augmentation strategies have been developed. The use of implantable scaffolds can help maintain the fibrin clot within the defect, facilitate cell adhesion and migration, and improve integration with the surrounding cartilage. Animal models and early clinical trials have shown promising results in terms of improving the quality of the repair tissue [77, 78]. Another approach is the use of growth factors to enhance the microfracture repair. Bone morphogenetic proteins (BMPs), such as BMP‑7 and BMP‑4 and cytokine modulation, specifically interleukin‑1 receptor antagonist (IL-1ra) have been investigated for their ability to promote chondrogenesis and improve the properties of the repair tissue [79–82]. Other techniques involve the combination of scaffold implants with cultured chondrocytes or the use of HA to further enhance the repair process [80].

Adipose-derived stem cells (ADSC) have the ability to differentiate into various cell types, including bone and cartilage [83]. They are easier to isolate in larger quantities with minimal donor site morbidity compared to bone marrow. ADSCs also exhibit a higher proliferation rate compared to BM-MSCs [83]. ADSCs can be isolated from fat through mechanical or enzymatic processes [84]. One mechanical method uses a fat-processing device (Lipogem) that isolates the cellular component of harvested autologous fat, generating micronized fat that can be injected into the joint [85]. Lipogems has demonstrated the ability to yield higher amounts of progenitor cells and MSCs compared to normal lipoaspirate [86]. Even though there are limited studies on Lipogems in hip treatment, they reported improved clinical outcomes with higher mHHS scores [87, 88]. No complications or difficulties with liposuction were reported in both studies. ADSCs offer a potentially safer and easier alternative to BM-MSCs for treating small acetabular chondral defects during hip arthroscopy; however, further studies are needed to determine the specific indications for each technique [86].

Articular cartilage injuries that are too large for microfracture can be treated with autologous chondrocyte implantation (ACI), a two-stage surgical technique that involves removing the damaged cartilage and microfracturing the defect before implanting previously harvested chondrocytes mixed with a bioabsorbable matrix [89]. Limited reports exist on the use of ACI in the hip, primarily due to difficulties with harvest and the need for a surgical procedure on an unaffected joint. However, Akimau et al. [90] described a case of ACI in a 31-year-old male with femoral head osteonecrosis, resulting in improved HHS and functional outcomes. Similarly, Fontana et al. [91] conducted a retrospective study comparing ACI to debridement, showing significantly better HHS outcomes in the ACI group after approximately 5 years of follow-up. However, the formation of viable cartilage was not confirmed in this study. These findings suggest that ACI may be a beneficial treatment option for chondral lesions in the hip, while arthroscopic debridement has limited utility, especially for larger lesions.

Autologous matrix-induced chondrogenesis (AMIC) is a surgical technique that involves the use of a type I/III collagen matrix in conjunction with microfracture to treat chondral defects of grades 3 and 4 that measure 2–4 cm^2^ [92]. During the procedure, the matrix is inserted into the joint using arthroscopy to cover the defect and stabilize the blood clot that results from microfracture, providing a framework for the formation of repair tissue [92]. Thier et al. [93] conducted a short-term study on arthroscopic injectable matrix-associated autologous chondrocyte implantation (MACI) for hip cartilage defects. Results showed improved patient outcomes in terms of activity level, quality of life, and pain reduction after a 19-month follow-up. Krueger et al. [94] evaluated the clinical outcome of arthroscopic matrix-associated injectable autologous chondrocyte implantation (ACI) for large acetabular cartilage defects. Findings revealed promising results with significant improvements in hip scores and subjective assessments after a 3-year follow-up, indicating the effectiveness of injectable ACI for weight-bearing zone defects.

The culture process involved in 3D-ACI generates redifferentiated autologous chondrocytes along with their extracellular matrix, resulting in scaffold-free 3D spheroids of neocartilage [95, 96]. These 3D constructs are injectable solutions, making the second step of chondrocyte implantation similar to injecting scaffolds into the defect site [95]. Studies evaluating the efficacy of 3D-ACI in the treatment of chondral defects in both the knee and hip have reported promising results [96, 97]. These investigations have demonstrated improved patient outcomes, such as increased mHHS and iHOT scores, and successful cartilage healing [97–99]. Even patients with larger defects have shown favorable results with the ease of application and adhesive properties of 3D-ACI [98]. While 3D-ACI appears to be a safe and effective treatment option for medium to large articular cartilage defects, further studies are required to assess its long-term benefits compared to the risks associated with longer culture times and the complexity of preparation, which may contribute to failure [95].

Autologous minced cartilage implantation (AMCI) has emerged as a promising technique for addressing acetabular cartilage lesions in patients with femoroacetabular impingement syndrome (FAIS). This innovative approach, described in recent studies by Zimmerer et al. [100] and Gebhardt et al. [101], involves arthroscopic preparation of the damaged cartilage, followed by mincing of autologous cartilage fragments using specialized instruments. These minced cartilage fragments, collected with the an autologous tissue collector (Graftnet™ system, Arthrex, Inc., FL, USA), are then augmented with autologous conditioned plasma (ACP) and implanted into the lesion site.

Osteochondral autograft transplantation (OAT) entails the transplantation of osteochondral plugs that are harvested from the nonweight-bearing surface to fill larger defects and is typically used when microfracture or other treatments have failed. On the other hand, mosaicplasty involves the transplantation of multiple smaller osteochondral plugs from a healthy articular surface to fill multiple smaller defects [102]. Mosaicplasty is often used in the knee and requires surgical hip dislocation when used in the hip. The technique has been utilized to address osteochondral defects in the femoral head, which has demonstrated the ability to enhance clinical outcomes and range of motion. The OAT can be performed either arthroscopically or through an open arthroscopic retrograde approach, depending on the placement of the defect. OAT has been shown to be efficacious in treating chondral lesions and osteonecrosis of the femoral head in the hip, leading to notable advancements in clinical scores. Recent studies have found that both mosaicplasty and OAT are effective in treating osteochondral defects, particularly in the femoral head. The studies report significant improvements in patient outcomes and pain relief with both procedures, although there may be a risk of subsequent hip arthroscopy with mosaicplasty. The disadvantages of OAT therapy include a relatively new procedure with limited long-term data, is not suitable for all, is not the treatment of choice for isolated full-thickness chondral defects at the hip, because of the unfavorable risk-benefit profile, can be technically demanding, and is not a permanent solution [102–107].

Osteochondral allograft transplantation (OCA) is a promising treatment option for osteochondral lesions of the hip, particularly for large defects that are difficult to treat with alternative techniques. OCA permits the replacement of a damaged joint surface with a single-stage technique that does not cause morbidity at the donor site. In addition, the application of OCA provides an instantly functional joint surface and can lead to the replacement of hyaline cartilage [108–110]. However, the survival of chondrocytes from the moment of graft procurement to the time of implantation can be affected by the length of storage time after graft procurement, with the survival of the graft being significantly diminished after 28 days of storage. Various studies have reported positive outcomes with the use of OCA transplantation for treating osteochondral defects in the hip joint. The use of fresh allografts has been found to avoid donor site morbidity, while the anterior approach allows faster rehabilitation and an earlier return to function. However, challenges with OCA transplantation include donor tissue matching, the timing of donation and implantation, limited supply of donor tissue, and potential nonunion or failure to transform into live tissue. Overall, while OCA transplantation may have advantages over other treatments, it is important to consider these challenges before deciding on a course of action [108–110].

Prosthetic biocomposites have emerged as a promising approach for the repair of osteochondral defects, offering potential solutions to the challenges associated with tissue regeneration. Several studies have explored the use of synthetic materials as scaffolds to guide tissue regeneration in osteochondral defect repair. One study by Frassica and Grunlan highlighted the importance of synthetic materials with instructive properties, which can influence cellular behavior and promote tissue growth [111]. They discussed the development of synthetic scaffolds with complex chemical and morphological features, prepared using various fabrication techniques, to restore both articular cartilage and underlying bone. Another review by Fu et al. summarized different scaffold types, such as porous, hydrogel, fibrous, and composite scaffolds, and evaluated their advantages and disadvantages in osteochondral tissue engineering [112]. They emphasized the challenges in achieving anatomical, biochemical, and biomechanical stratification in tissue regeneration. Additionally, Xu et al. focused on the construction of a bilayered composite scaffold using chitosan and chitosan-beta-tricalcium phosphate, which demonstrated chondrogenic and osteogenic abilities, leading to effective repair of osteochondral defects in a rat model [113].

## Surgical recommendations

El Bitar et al. developed a straightforward algorithm to assist with decision-making in patients presenting with symptoms of full-thickness femoral head and acetabular lesions, given the wide range of surgical treatment options available. This algorithm can be helpful in planning surgical interventions for chondral lesions of the hip ([114]; Table 4).

**Table 4:** Summary of proposed treatment algorithm by El Bitar et al.

Lesion size	Femoral head	Acetabulum
< 2 cm^2^	1st line: microfracture, Cartilage repair	Microfracture
	2nd line: mosaicplasty, OCA transplantation	
2–6 cm^2^	Microfracture, osteochondral allograft transplantation	Microfracture
6–8 cm^2^	Total hip arthroplasty, osteochondral allograft	Total hip arthroplasty
	Transplantation	
> 8 cm^2^	Total hip arthroplasty	Total hip arthroplasty

## Guidelines by the DGOU group for biologic reconstruction of full sized cartilage defects of the hip

The German Society of Orthopedics and Trauma (Deutsche Gesellschaft für Orthopädie und Unfallchirurgie, DGOU) has published guidelines for the biologic reconstruction of full-sized cartilage defects of the hip [115]. The guidelines were developed by the DGOU’s Group for Clinical Tissue Regeneration and the Hip Committee of the working group for arthroscopy and joint surgery (AGA) [115]. The guidelines recommend the treatment options for full-sized cartilage defects of the hip given in Table 5.

**Table 5:** DGOU group’s guidelines

Aspect	Treatment guidelines
Cartilage defect size	Matrix-assisted autologous chondrocyte transplantation (MACT) is preferred for full-thickness cartilage defects > 1.5–2 cm^2^
	Minimally invasive MACT (e.g., injectable chondrocyte implants) favored in the hip joint
	Bone marrow-stimulating technique + biomaterial preferred for cases not suitable for MACT
	Single-stage procedure may be considered for lesions smaller than 1.5–2 cm^2^
Age limit for surgery	No definitive upper age limit for joint-preserving surgery or MACT due to biological variability
Stage of the disease	Advanced hip osteoarthritis contraindicates hip-preserving surgery

## Postoperative management

Hip preservation surgery often involves a combination of procedures, and the postoperative rehabilitation plan should consider all concurrent disorders [116]. Chondroplasty generally does not require any postoperative restrictions, while microfracture procedures necessitate weight-bearing precautions to protect the affected area [116]. The duration of weight-bearing restrictions after microfracture can range from 2 to 8 weeks, after which patients gradually increase their weight-bearing to full weight [116]. Other procedures, such as AMIC/ACI, mosaicplasty, osteochondral transplantation, and articular cartilage repair, usually necessitate 6 weeks of touch-down weight bearing followed by 6 weeks of partial weight bearing [117]. However, rehabilitation protocols may vary depending on the surgeon’s preferences and the patient’s specific condition. Recent studies have suggested that immediate weight-bearing after microfracture may not compromise clinical outcomes [118, 119]. A systematic review found that weight-bearing restriction after microfracture may not provide additional benefits and that early rehabilitation may be beneficial for postoperative outcomes [119].

## Conclusion

Osteochondral injuries in the hip are debilitating conditions that can significantly impair daily life and negatively impact the quality of life. These injuries often result in progressive joint damage, leading to end-stage osteoarthritis. Treating such injuries is especially challenging in young and active patients because the hip joint regularly handles significant stresses through an only weight-bearing compartment. To address these injuries, various strategies are used to repair or reconstruct chondral/osteochondral tissue. These include biological therapies (stem cells, scaffolds or cell-based therapies) which have shown promise in promoting healing and regeneration of damaged tissue. Realignment procedures surrounding the hip joint are also commonly necessary to optimize outcomes. Surgical procedures such as osteotomies and arthroscopies may be used to address these issues. While various treatment options have shown success, including repair, microfracture, autograft chondrocytes, and allograft transplants, there is still a lack of head-to-head comparisons and large sample sizes in the literature. Therefore, further research is needed to evaluate the efficacy of different treatments for managing chondral injuries of the hip joint and to develop appropriate clinical guidelines for patient care. Early detection and prompt management of these injuries are crucial to prevent irreversible joint damage and minimize the need for invasive surgical interventions. A multidisciplinary approach involving orthopedic surgeons, radiologists, and rehabilitation specialists is often required to achieve the best outcomes for patients with chondral/osteochondral injuries of the hip.

## Abbreviations

3D-ACI: Three-Dimensional Autologous Chondrocyte
ACEA: Anterior center-edge angle
ACI: Autologous chondrocyte implantation
ACT: Autologous chondrocyte transplantation
ADSCs: Adipose-derived stem cells
AGA: Arbeitsgemeinschaft für Arthroskopie und Gelenkchirurgie (German working party for arthroscopy and joint surgery)
AI: Acetabular index
ALAD: Acetabular labrum articular disruption
AMIC: Autologous matrix-induced chondrogenesis
ARCO: Association Research Circulation Osseous classification
AVN: Avascular necrosis
BMAC: Bone marrow aspirate concentrate
BM-MSC: Bone marrow mesenchymal cells
CEA: Center-edge angle
CT: Computed tomography
DDH: Developmental dysplasia
DGEMRIC: Delayed gadolinium-enhanced magnetic resonance imaging of cartilage
DGOU: Deutsche Gesellschaft für Orthopädie und Unfallchirurgie (German Society of Orthopedics and Trauma)
FAI: Femoroacetabular impingement
HA: Hyaluronic acid
ICRS: International Cartilage Repair Society
iHOT: International hip outcome tool
IL-1ra: Interleukin-1 Receptor Antagonist
LCEA: Lateral center-edge angle
LCP: Legg-Calvé-Perthes
MACT: Matrix-assisted autologous chondrocyte transplantation
MATT: Microfragmented adipose tissue transplantation
MMHS: Modified Harris hip score
MRI: Magnetic resonance imaging
MSCs: Mesenchymal stem cells
NSAIDs: Nonsteroidal anti-inflammatory drugs
OA: Osteoarthritis
OAT: Osteochondral autograft transfer
OCA: Osteochondral allograft transplantation
OCD: Osteochondritis dissecans
OCL: Osteochondral lesion
PRP: Platelet-rich plasma
SCFE: Slipped capital femoral epiphysis

## Declarations

For this article, no studies with human participants or animals were performed by any of the authors. All studies mentioned were in accordance with the ethical standards indicated in each case.
